# Just watching is not enough: Fostering simulation-based learning with collaboration scripts

**DOI:** 10.3205/zma001181

**Published:** 2018-08-15

**Authors:** Jan M. Zottmann, Peter Dieckmann, Tatjana Taraszow, Marcus Rall, Frank Fischer

**Affiliations:** 1 Klinikum der LMU München, Institut für Didaktik und Ausbildungsforschung in der Medizin, Munich, Germany; 2 Herlev Hospital, Center for Human Resources Capital Region of Denmark, Copenhagen Academy for Medical Education and Simulation (CAMES), Herlev, Demark; 3 University of Copenhagen, Department for Clinical Medicine, Copenhagen, Denmark; 4 University of Stavanger, Faculty of Health Sciences, Stavanger, Norway; 5 Humboldt-Universität zu Berlin, Institut für Erziehungswissenschaften, Berlin, Germany; 6 InPASS, Institut für Patientensicherheit und Teamtraining GmbH , Reutlingen, Germany; 7 LMU München, Lehrstuhl für Empirische Pädagogik und Pädagogische Psychologie, Munich, Germany

**Keywords:** simulation training, observational learning, instructional support, collaboration scripts, crisis resource management

## Abstract

**Aims:** In addition to medical facts, medical students participating in simulation-based training are supposed to acquire general knowledge, e.g. heuristics to cope with critical incidents. While active participation is considered a major benefit of this kind of training, a large portion of students’ time is often spent observing peers acting in the simulator. Thus, we instructionally supported learners with a collaboration script (i.e., a set of scaffolds that distribute roles and activities among learners in group learning situations) during observational phases of a simulation-based training. Our script was designed to help learners focus on heuristics and to facilitate more (inter-)active participation. We hypothesised that scripted learners would benefit from the instructional support with respect to individual and collaborative learning processes as well as individual learning outcomes.

**Methods: **Thirty-four medical students in their 7^th^ to 12^th^ semester participated in this field study with control group design. The independent variable was the collaboration script (with/without). Four voluntary emergency courses with a full-scale simulator were examined. The acquisition of skills related to Crisis Resource Management (CRM) heuristics was one of the learning goals of these courses. The collaboration script induced learners to perform specific activities during and after each observational phase of the training. Further, the script sequenced the order of activities and assigned roles to the learners. Learning processes were measured on an individual level (by means of notes taken by learners during observational phases) and on a collaborative level (by means of learners’ comments). Learning outcomes were measured with pre- and post-self-assessment of CRM skills and a brief video-based CRM skills test at the end of the course.

**Results: **The collaboration script had the expected positive effect on individual and collaborative learning processes, leading to an increased focus on heuristic strategies and increased collaborative activity of scripted learners. There was no evidence that the experimental conditions differed regarding the objective measure of individual learning outcomes. However, self-assessment data revealed that students in the control condition perceived a higher improvement of CRM skills throughout the course. We suggest that our script might have helped learners adjust an illusion of their own competency – such an illusion may have appeared in the control group as a result of processing fluency.

**Conclusions: **Findings suggest that simulation-based training in medical education can be enhanced with additional instructional support in the form of collaboration scripts designed to turn observational course phases into more active and better focused learning experiences.

## Zusammenfassung

**Ziele: **Medizinstudierende, die an einem simulationsbasierten Training teilnehmen, sollen zusätzlich zu medizinischem Faktenwissen auch allgemeineres Wissen wie Heuristiken für den Umgang mit kritischen Ereignissen erwerben. Obgleich die aktive Teilnahme als großer Vorteil dieser Art von Training angesehen wird, verwenden die Studierenden dabei einen Großteil ihrer Zeit darauf, die im Simulator handelnden Personen zu beobachten. Vor diesem Hintergrund wurden Lernende während der Beobachtungsphasen eines simulationsbasierten Trainings instruktional mit einem Kooperationsskript (d. h. einem Set von Scaffolds, die Lernenden in Gruppen bestimmte Rollen und Aktivitäten zuweisen) unterstützt. Das Skript zielte darauf ab, die Lernenden bei der Fokussierung auf Heuristiken zu unterstützen und eine (inter-)aktivere Kursteilnahme zu fördern. Der Studie lag die Hypothese zugrunde, dass die Lernenden von einer instruktionalen Unterstützung mit Kooperationsskript sowohl im Hinblick auf individuelle und kooperative Lernprozesse als auch auf den individuellen Lernerfolg profitieren würden.

**Methoden: **Insgesamt nahmen 34 Medizinstudierende im 7. bis 12. Semester an dieser Feldstudie mit Kontrollgruppendesign teil. Die unabhängige Variable war der Einsatz des Kooperationsskripts (mit/ohne). Es wurden vier simulationsbasierte Notfallkurse untersucht, an denen die Studierenden freiwillig teilnahmen. Der Erwerb von Fähigkeiten zur Anwendung der Prinzipien des Crisis Resource Management (CRM) war ein Lernziel dieser Kurse. Das Kooperationsskript instruierte die Lernenden, während der Beobachtungsphasen des Trainings und im Anschluss bestimmte Aktivitäten auszuführen. Darüber hinaus legte das Skript die Reihenfolge der Aktivitäten fest und wies den Lernenden Rollen zu. Individuelle Lernprozesse (gemessen anhand der während der Beobachtungsphasen gemachten Notizen) und kooperative Lernprozesse (gemessen anhand von Äußerungen während der Kooperations- und Beobachtungsphasen) wurden erfasst. Der Lernerfolg wurde zu Beginn und am Kursende über eine Selbsteinschätzung der Fähigkeiten zur Anwendung von CRM-Heuristiken gemessen. Am Kursende erfolgte zusätzlich ein kurzer videobasierter CRM-Anwendungstest.

**Ergebnisse: **Das Kooperationsskript hatte den erwarteten positiven Effekt auf individuelle und kooperative Lernprozesse. Lerndende mit Skript zeigten eine erhöhte Fokussierung auf heuristische Strategien sowie erhöhte kooperative Aktivität. Die beiden experimentellen Bedingungen unterschieden sich jedoch nicht bezüglich des objektiven Maßes für individuellen Lernerfolg. Allerdings zeigten die Selbsteinschätzungsdaten, dass Studierende der Kontrollbedingung über den Kursverlauf hinweg einen größeren Zuwachs an Fähigkeiten zur Anwendung heuristischer Strategien wahrnahmen. Wir vermuten, dass unser Skript eine Kompetenzillusion auf Seiten der Lernenden verhinderte, die in der Kontrollgruppe als Ergebnis flüssiger Informationsverarbeitung aufgetreten sein könnte.

**Schlussfolgerungen:** Die Ergebnisse der Studie zeigen, dass simulationsbasierte Trainings in der medizinischen Ausbildung von einer zusätzlichen instruktionalen Unterstützung profitieren können – Beobachtungsphasen in solchen Trainings lassen sich mit Hilfe von Kooperationsskripts aktiver und zielgerichteter gestalten.

## 1. Introduction: Structuring joint observation in simulation-based training with collaboration scripts

Active participation is considered to be a major benefit of simulation-based training [1]. However, in many simulation-based training courses only few persons can act hands-on concurrently in the simulator, whereas the remaining students observe the ongoing scenario either live or via video. With this set-up, most of the individual learners’ time is, in fact, spent watching others perform. In these observational phases, formal guidance of learners is often lacking, as is the structure to use fellow observers as sources of information or validation of the learner’s own observations. Consequently, an important question is: How are such phases of observation spontaneously used by learners and how can they be instructionally supported for more effective learning? Another consideration concerns knowledge acquisition. It takes more than medical factual knowledge to be successful as a health professional; simulation-based courses provide learners with rich and dynamic situations in which more general knowledge, such as heuristics to cope with critical situations in health care settings, can be obtained in addition to domain-specific medical factual knowledge. The principles of Crisis Resource Management (CRM) are typical examples of medical-related heuristics. The concept of CRM [2] aims to help with coordinating and utilising all available resources to optimise patient safety. The CRM concept goes beyond team and communication skills – it covers individual cognitive aspects, such as fixation errors, allocation of attention, and anticipation. CRM heuristics comprise a total of 15 principles (including “call for help early”, “know the environment”, or “communicate effectively”, for instance) [3]. There is a lack of systematic empirical research on how students acquire knowledge about CRM heuristics in simulation-based training. This article presents an interdisciplinary field study aimed at

- instructionally supporting learners in a simulation-based training to focus on heuristic strategies for complex, dynamic situations; and
- designing observational phases for more effective learning processes.

In medical education, simulators provide controlled and safe practice opportunities, going beyond the learning possibilities in the clinical world: Simulations can be sped up, slowed down, or even stopped, for example, and the participants can take on roles and tasks they could not perform in clinical practice [4]. The BEME review by Issenberg and colleagues [5] notes that simulation-based medical education is best employed to prepare learners for real patient contact. The authors conclude that high-fidelity medical simulations are educationally effective. However, the question of how groups of learners use observational phases in simulation-based training for learning has not yet been the focus of systematic research. Decades of research on observational learning have shown that observation can dramatically facilitate the acquisition of concepts and principles as well as strategies for problem solving. Various studies have identified and evidenced the conditions under which observational learning is effective [e.g. [6]]. Theories like *Social Learning Theory* [7] and *Cognitive Apprenticeship* [8] suggest that substantial learning can take place by observing a model performing a simulation task. However, learning from a model requires several preconditions, such as focussing attention on the model’s crucial activities and having access to heuristic strategies that can be applied in a given scenario. 

This article proposes the use of collaboration scripts to instructionally support students in observational phases. Collaboration scripts are sets of scaffolds that distribute roles and activities among learners in group learning situations. These scripts help to structure the learning processes by introducing a sequence for collaborative activities [9], [10]. Scripts can also facilitate collaborative activities, such as exchanging new ideas (externalising), asking questions (elicitating), or negotiating (consensus building) with learning partners [11] Moreover, scripts support deeper individual elaboration [12] and better coordination of collaborative knowledge-building activities [13], leading to better individual learning outcomes [14]. Only few empirical studies have investigated collaboration scripts in the context of medical education in general and simulation-based learning in particular. Rummel and Spada [15] found positive effects of scripted collaborative problem-solving when examining dyads of physicians and psychologists who collaborated remotely in a computer-mediated environment to solve a complex clinical case that required an interdisciplinary solution. Stegmann and colleagues [16] found that medical students who observed peers in simulated doctor-patient communication substantially benefitted from instructional support directing the students’ attention to specific aspects of the simulation. Kiesewetter and colleagues [17] examined the internalization of collaboration scripts (i.e., fast retrieval of script information without external instructional support) in medical education. Our study aimed to contribute to the growing body of evidence for the effectiveness of collaboration scripts in real educational settings and, more specifically, the context of medical education. 

## 2. Research questions and hypotheses

This field study investigated the effects of collaboration scripts in observational phases of simulation-based courses on individual learning processes (RQ1), collaborative learning processes (RQ2), and individual learning outcomes regarding the application of CRM heuristics (RQ3). 

The following hypotheses were tested:

1. The collaboration script has a positive effect on individual learning processes (particularly on the elaboration of CRM heuristics);
2. the collaboration script has a positive effect on the content (i.e., higher frequency of reference to CRM heuristics) as well as the type of activities (i.e., higher frequency of externalisation, elicitation, and consensus building) in collaborative learning processes;
3. the collaboration script has a positive effect on individual learning outcomes with respect to the ability to apply the CRM heuristics in subsequent simulated emergency situations.

## 3. Methods

### 3.1. Participants and design

Thirty-four medical students in their 7^th^ to 12^th^ semester at Tuebingen University, Germany, participated in the study (average age: 25.75 years). We examined a convenience sample of four simulation-based emergency courses. Each course consisted of four consecutive training days and included a dedicated one-hour lecture on CRM heuristics on the second training day. We randomly assigned students to one of two courses with the independent variable being the use of a collaboration script. The participants in each course did not differ with respect to age, gender, and prior knowledge. Fourteen students indicated they had participated a simulation-based training before, but none of them had done so more than once. Participation in the course was voluntary. Participants were informed about the empirical study being conducted during the course, but they were not made aware of their assignment to a certain experimental condition. This study was carried out in accordance with the Declaration of Helsinki of the World Medical Association (WMA). The guidelines for safeguarding good scientific practice of the German Research Foundation (DFG) applied in addition.

#### 3.2. Description of the simulation-based training

**Figure 1:**
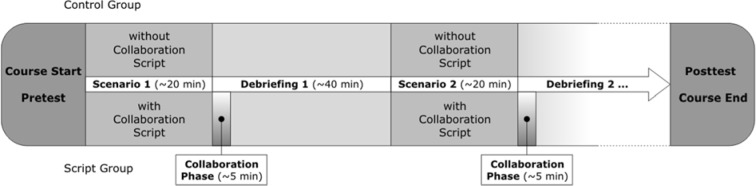
Procedure of the simulation-based training for both experimental conditions.

A patient simulator (Laerdal SimMan®) was embedded into a typical clinical setting with authentic, fully functional medical equipment and treated similar to an actual patient. At the beginning of each course, participants took a CRM pretest that asked them to rate their ability to apply CRM heuristics in an emergency situation. After that, the instructor informed the participants about the simulator and the procedures of the scenario and the debriefing. The simulation-based training consisted of seven or eight scenarios, each simulated emergency situations with durations of about 20 minutes, in which a team of four to five students handled an incident and rescued the patient. The remaining six to eight students in the course observed the actions taking place in the simulator from a room nearby via audio/video projection. At the beginning of each observational phase, learners received instructions according to the experimental conditions (two courses with collaboration scripts, two courses without). After completion of each scenario, a video-assisted debriefing of about 40 minutes took place in which the instructor together with the whole group analyzed and reflected on the simulated situation. In each scenario, a different team of students was working in the simulator, so that all course participants had encountered one or two hands-on experiences and numerous observations by the end of the training. Scenarios were not completely identical across the four courses, but they were all directed toward these aims:

- Learners should be put into typical urgent situations in order to practice and to get used to the stress level of real-life emergencies.
- Learners should be given opportunities to obtain and apply domain-specific knowledge (i.e., medical factual knowledge).
- Learners should be given opportunities to obtain and apply more general knowledge (i.e., CRM heuristics). The completion of the last debriefing was followed by an individual CRM posttest that paralleled the individual pretest (see figure 1 (Fig. 1)).

#### 3.3. Experimental conditions 

##### 3.3.1. Control condition

The learners in the control condition (*n*=20) experienced a regular simulation-based training without a collaboration script. Students in this condition were given a sheet of paper to take notes during the observational phases. The paper contained the following task description: “Observe your fellow students in the simulator. You may take notes on this sheet”. The students did not receive any additional instructions regarding collaboration during or after the observational phases.

##### 3.3.2. Collaboration script condition

**Figure 2:**
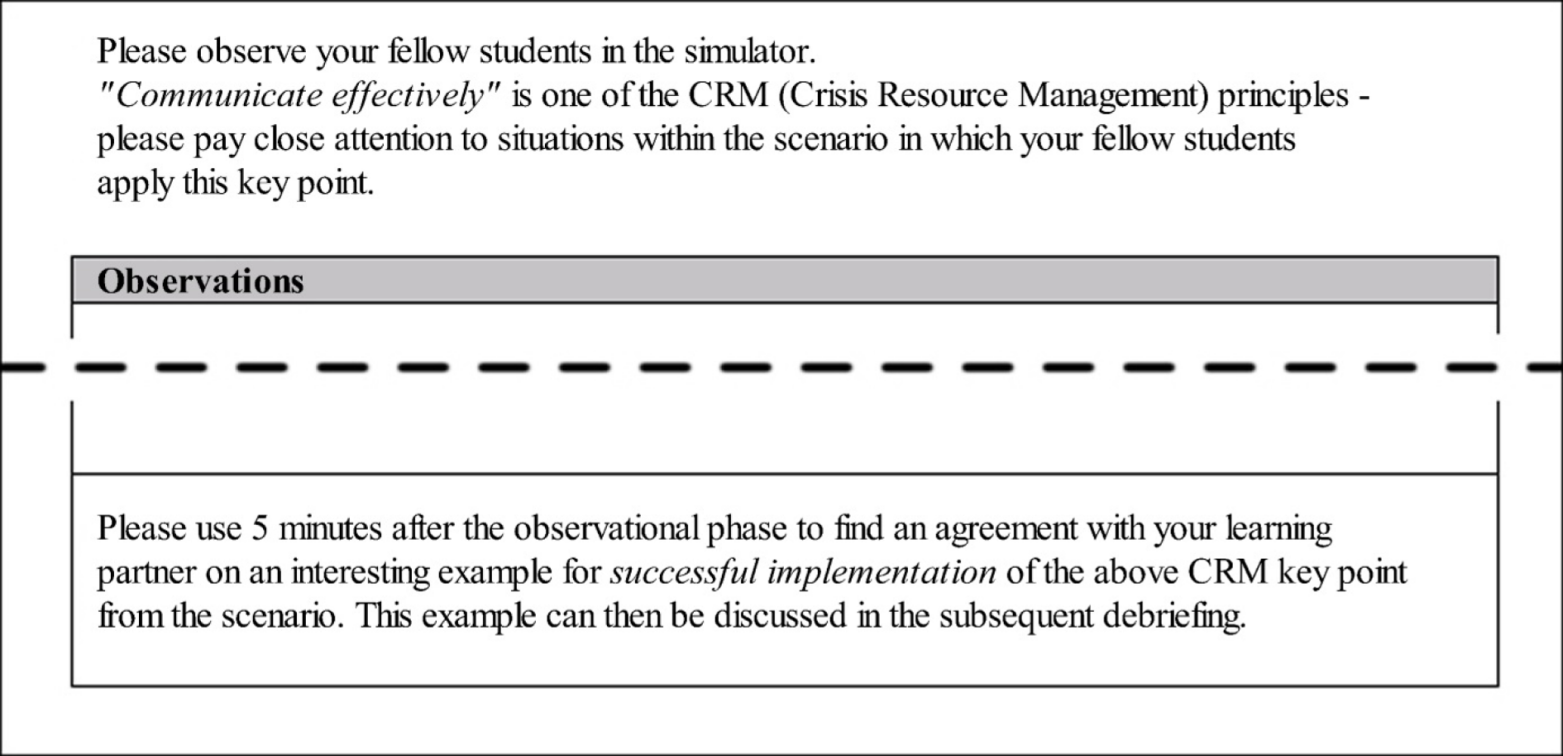
The collaboration script as distributed during the study (in this example, the learner was asked to focus on the CRM principle “communicate effectively” and take over the role of the analyst for positive situations in the collaboration phase).

The collaboration script in this study induced the learners in the script condition (*n*=14) to perform specific activities, assigned an order to these activities, and also assigned roles to the learners [10]. The script design structured individual learning processes during the observational phases by drawing the students’ attention to specific CRM heuristics during the observation. Each student in this group was given the task to observe the students in the simulator on the basis of a single CRM principle and to take notes. The principles to be observed varied throughout the observational phases; subgroups of two learners were provided with collaboration scripts focusing on the same CRM principle. The collaboration script also assigned roles to each of the two learners: The *analyst for positive situations* had to pay attention during the scenario to situations in which fellow students successfully applied the CRM principle and present those situations during the collaboration phase. In contrast, the *analyst for negative situations* had to focus on situations in which CRM principles were not applied or were applied in an erroneous fashion and report on those. During the collaboration phase, learners in each dyad had to jointly decide on one interesting example of a successful implementation as well as one of a less successful implementation of their respective CRM principle from the scenario (see figure 2 (Fig. 2)). In order to allow for a change of perspectives, leading to deeper elaboration and more comprehensive understanding of the content [9], learners had to switch roles after each scenario. Each individual phase of observation lasted about 20 minutes, and the following collaboration phase took 5 minutes.

#### 3.4. Data sources, dependent variables, and instruments

##### 3.4.1. Individual elaboration

We measured the individual elaboration by means of notes taken by the learners during the observational phases. We distinguished medical content from content regarding heuristic CRM strategies. Segments containing medical factual knowledge only (e.g., “check pupils regularly”) received a “medicine-related” code. A segment was coded as CRM-related if the note mentioned interpersonal actions (e.g., “team communicates”), resembled specific CRM principles (e.g., “called for help”), or if the note was about the practical implementation of a CRM principle in a particular situation (e.g., “precise instruction to nurse”). The frequency of the learners’ notes was counted. As an indicator of individual elaboration, we used the sum of all segments referring to medical content and to CRM heuristics, which could be identified in the individual note sheets during the observational phase. We examined 16 out of 30 phases of observational learning that took place within the courses, resulting in a data source of 92 individual pages of handwritten notes. The coding of the segments corresponded sufficiently between two independent raters (Cohen’s *κ*=.91).

##### 3.4.2. Collaborative elaboration

We measured collaborative elaboration by using audio recordings of the learners’ comments made during the collaborative phases and their exchanges during the observational phases. Regarding collaborative elaboration, we examined 11 out of 30 phases for the collaborative and the observational learning phases, which equal 55 minutes out of 150 of the collaboration phases and 55 minutes out of 600 of the observational phases. Two independent raters analysed these comments using a time-sampling procedure [11] as well as interval sequences [18] with respect to content and activity. The raters distinguished between medical factual content and content regarding CRM heuristics; the classification of the comments regarding the content corresponded sufficiently between the two raters (Cohen’s *κ*=.74 to .93; *MD*=.77). Beyond content, the raters coded the type of collaborative activity by classifying comments along the categories of elicitation, externalisation, and consensus building [11]. The correspondence between the two raters concerning the classification of the comments in these categories was Cohen’s *κ*=.72.

In addition, we asked learners to fill in a collaboration questionnaire with 14 items (e.g. “I discussed CRM principles with others during the observational phases”) at the end of the training. Students retrospectively ranked their interaction on a four-point Likert scale ranging from 1 (I don’t agree) to 4 (I agree). An individual mean total score was used as an indicator for the self-assessed activity (i.e., the perceived intensity of information exchange) during the observational phases. The reliability of this scale corresponded satisfactorily, with Cronbach’s *α*=.67.

##### 3.4.3. Knowledge acquisition

*Subjective measure:* The application of heuristic strategies in a crisis situation was measured with pre- and post-self-assessment of CRM skills. In the pretest, we asked our participants to evaluate their expertise in handling emergency situations with 15 items on a four-point Likert scale. Each of the items referred to one of the 15 CRM principles (e.g., “how would you rate your ability to communicate effectively in an emergency situation?”). The reliability of the pretest was satisfactory (Cronbach’s *α*=.85). In the posttest questionnaire, we asked the learners again to rate their expertise in applying the CRM heuristics in an emergency (Cronbach’s *α*=.85). We used the mean of all posttest items adjusted for the pretest scores (calculated by means of regression analysis) as an indicator of self-assessed CRM skills.

*Objective measure:* We asked learners to apply CRM heuristics to a brief posttest video case depicting a situation in which a patient needed to be resuscitated. This case had been specifically designed and developed by medical experts to demonstrate the implementation of CRM (mainly non-successful) to students. The performance of the learners was used as an objective indicator for CRM skills. Learners had to analyze the behavior of the acting persons against the background of the CRM heuristics. A medical expert (i.e., experienced trainer of simulation-based courses) rated the learners’ written analyses and assigned grades from 1 (excellent) to 6 (failed) to them. 

##### 3.4.4. Statistical tests applied

We used t-tests for independent samples to determine the significance of the effects of the collaboration script towards individual and collaborative elaboration, as well as knowledge acquisition. We used an *α*-level of .05 for all statistical tests. 

## 4. Results

### 4.1. Individual elaboration (RQ1)

We found no initial differences between the two groups with respect to self-assessment data in the pretest, *t*(32)=-1.02, *p*=.317, n.s.. Regarding the individual elaboration of CRM heuristics during the learning phase, however, we found a significant difference between the script condition and the control condition, *t*(13)=-4.13, *p*=.001, *d*=1.73. None of the notes from the learners in the control condition group contained segments that could be coded as heuristic strategies. The two groups did not differ significantly regarding the individual elaboration of medical factual knowledge, *t*(32)=-0.35, *p*=.729, n.s. (see table 1 (Tab. 1) for an overview of the findings of the study).

#### 4.2. Collaborative elaboration (RQ2)

##### 4.2.1. Content

Regarding the content of the collaborative elaboration, a significant difference existed between the script learners and the control condition learners, with the latter not referring at all to CRM heuristics during the collaborative phases examined, *t*(12)=5.63, *p*<.001, *d*=1.80. Regarding medical factual knowledge, we found no significant difference between the script condition and the control condition, *t(*16)=0.47, *p*=.645, n.s.. 

##### 4.2.2. Activities: Elicitation, externalisation, consensus building

Scripted learners generally participated more actively than learners from the control condition. Regarding elicitations, learners who were supported with the script asked significantly more questions than did learners in the control condition, *t*(16)=2.47, *p*=.025, *d*=1.30. Regarding externalisations, learners supported with the script shared significantly more thoughts with their peers than did learners in the control condition, t(15.96)=2.66, p=.017, d=0.97. Regarding consensus building, scripted learners were more involved in negotiation processes than were learners in the control condition, *t*(15.81)=4.63, *p*<.001, *d*=1.63. In addition, subjective measures from the collaboration questionnaire revealed that learners in the scripted group also felt they had been more active in terms of exchanging information related to heuristic strategies than learners in the control group. This difference in self-assessed activity was significant, *t*(31)=-2.31, *p*=.028, *d*=0.82.

#### 4.3. Knowledge acquisition (RQ3) 

##### 4.3.1. CRM skills test

There was no significant difference between the experimental conditions regarding performance in the heuristic strategies skills test. Learners in both conditions, with and without script, performed slightly below average, according to the expert rater, *t*(28.97)=0.47, *p*=.642, n.s.

##### 4.3.2. Self-assessment of CRM skills

Regarding the effect of the collaboration script on the self-perceived improvement of heuristic strategy skills, we found a difference between scripted learners and learners in the control condition. Learners in the control condition perceived a significantly higher gain of CRM skills throughout the course than did scripted students, *t*(31)=2.07, *p*=.047, *d*=0.74.

## 5. Discussion

Results show that the collaboration script had the expected positive effects on processes of collaboration and partly on learning. With regard to the content, scripted students showed an increased focus on heuristic strategies and improved information exchange with the heuristics as compared to the control condition. The overall behaviour of the scripted students during the observational phases can be described as more mindful [19]. The scripted students showed increased activity with regard to elicitation, externalisation, and consensus building. While we cannot be sure that the student’s notes and comments contained *all* of their thoughts on CRM heuristics, our findings suggest that this aspect was rarely considered in the control condition – a condition that represented typical simulation-based training setups in practice. However, we found no statistical difference between the two conditions regarding the elaboration of medical factual knowledge. Apparently, the shifting of the students’ focus on CRM did not occur at the cost of factual knowledge acquisition.

Regarding individual learning outcomes, subjective and objective measures diverged strongly. Interestingly, the self-assessment data revealed that students in the control condition actually perceived a higher improvement of their skills to apply heuristic strategies throughout the course than did the scripted learners. This could be evidence for the low ability of medical students to assess their own skills appropriately [20]. We argue, however, that it is more likely that the collaboration script helped learners adjust an illusion of their own competency [21]. Such an illusion may have appeared in the control group as a result of processing fluency [22]: Learners who experience information processing as being easy and fluent are in danger of basing inadequate judgments on this experience. For example, studies have shown that fluent processing increases the perceived fame of non-famous names [23] or the perceived truth of repeated assertions [24]. Likewise, empirical studies have demonstrated that the subjective experience of ease may lead learners to a positively biased self-assessment of performance [e.g. [22]]. It seems plausible that participants in the control group, who were not instructionally supported to focus on CRM heuristics in the observational phases, perceived the concept of CRM as easily understandable – probably even more so when compared to medical factual knowledge. Subsequently, students in the control group may have considered the acquisition of CRM skills an easy task. Scripted learners, in contrast, invested more effort in the elaboration of CRM heuristics throughout the training. Hence, one could assume the use of the scripts counteracted a negative effect of processing fluency by making the learning experience *more difficult* [25], thus enabling learners to assess their skills more accurately.

Even though individual and collaborative learning processes could be fostered, the script failed to show the expected positive effect on learning outcomes. This finding is in contrast to previous research that demonstrated a clear connection between deeper cognitive elaboration induced by scripts and improved learning outcomes [12], [26]. A possible explanation for this discrepancy could be that strategy training is known to sometimes have initially negative effects on performance when new learning strategies are adopted [27]. A positive effect of the script could have been neutralised by such a deficiency, and the measurement of the effects might have come too early in this adaptation process. A second explanation could be that the intervention was too short to find an effect on learning outcomes. The script was applied to only some parts of the training, whereas collaboration scripts in other studies were structuring the learning processes for higher shares of the total learning time [13]. A third explanation for the missing effect could refer to the way in which the application of CRM was assessed: An often-articulated request in research on simulations is that newly acquired skills should be evaluated in tests of application and transfer [28]. Perhaps the analysis of a short video case was not a fully valid way to assess students’ skills. Different learning styles and preferences in our study participants [cf. [29]] may eventually have had an impact on learning processes and outcomes as well. The relatively small number of participants is another limitation of this study. Further research with larger samples, including both longer periods of collaboration script intervention and delayed measurement of the application of the newly learned strategies, will help to decide between the alternative explanations outlined above. As an option, course participants could evaluate a video of their own performance in the simulator as a CRM skills test at the end of the course.

Other aspects of simulation-based training might benefit from instructional support as well. For instance, collaboration scripts could help in making video debriefings more effective by clarifying the roles of those involved and empowering participants to become partners in the learning process rather than merely being learners who are taught [30]. Debriefing is seen as an important part of simulation-based learning as it stimulates reflection about the actions during the scenario [31]: Participants can think about what they did well in the scenario and how they can reproduce this good performance, but they can also consider aspects that would further improve their performance [32]. Such reflection would certainly benefit from techniques to support it in a way that matches the learning goals. Collaboration scripts might be a way to foster reflexivity during debriefings, a concept that is focused increasingly [33]. 

In conclusion, our findings suggest that simulation-based courses in medical education can be improved substantially with additional instructional support. Such support appears to be necessary when learners are supposed to acquire knowledge, skills, and attitudes needed for safe practice within this scope. Collaboration scripts can help to turn observational phases in training into more (inter-)active, more mindful, and better focused experiences. Our results indicate that scripts can also support learners to self-assess more accurately their progress toward skill acquisition, thus contributing to a crucial aspect of simulation-based learning, namely, providing educational feedback [5], [30]. However, we suggest replication to further validate our findings in this regard. Our results further emphasise the need to investigate the social dimension of simulation-based training more systematically [34]. On a more general level, these results provide further evidence that collaboration scripts do show positive effects, not only in the laboratory but in real educational contexts as well. 

## Competing interests

The authors declare that they have no competing interests. 

## Figures and Tables

**Table 1:**
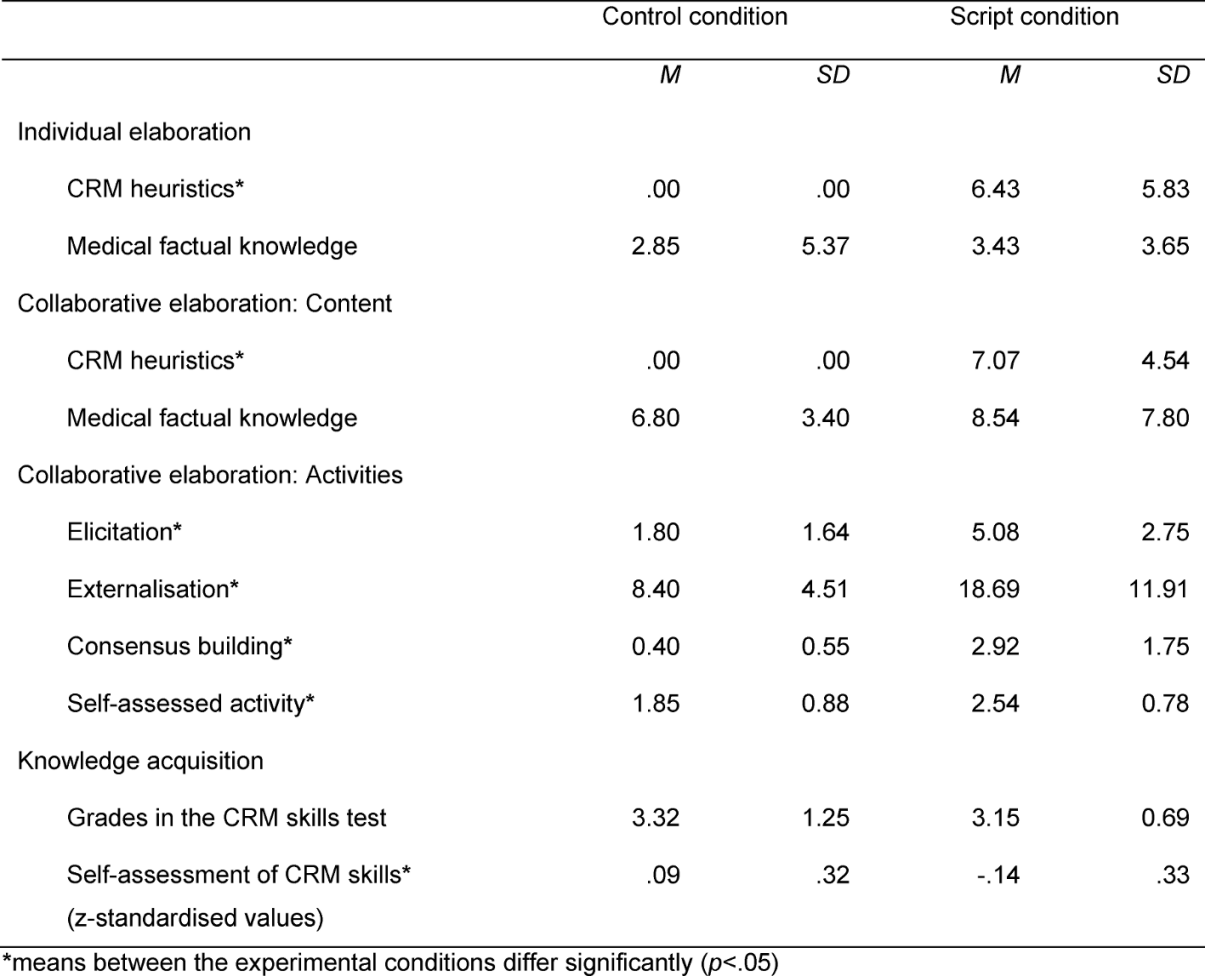
Findings regarding individual elaboration (measured by number of notes taken during observational phases), collaborative elaboration (measured by number of comments made during collaborative and observational phases), and knowledge acquisition (measured by grades in the CRM skills test and self-assessed CRM skills in the posttest). Presented are means (M) and standard deviations (SD).
